# Intention to seek emergency medical services during community overdose events in British Columbia, Canada: a cross-sectional survey

**DOI:** 10.1186/s13011-022-00484-0

**Published:** 2022-07-26

**Authors:** Bradley Kievit, Jessica C. Xavier, Max Ferguson, Heather Palis, Soroush Moallef, Amanda Slaunwhite, Terri Gillis, Rajmeet Virk, Jane A. Buxton

**Affiliations:** 1 School of Population and Public Health, University of British Columbia, 2206 E Mall, Vancouver, BC V6T 1Z8 Canada; 2 British Columbia Centre for Disease Control, 655 W 12th Ave, Vancouver, BC V5Z 4R4 Canada; 3 Department of Psychiatry, University of British Columbia, 2255 Wesbrook Mall, Vancouver, BC V6T 1Z3 Canada; 4 British Columbia Centre on Substance Use, St. Paul’s Hospital, Vancouver, BC Canada; 5 Faculty of Health Sciences, Simon Fraser University, Burnaby, BC Canada; 6 Métis Nation British Columbia, 13401 108th Ave, Surrey, BC V3T 5T3 Canada

**Keywords:** 911-calling, Drug overdose, Good Samaritan law, Emergency medical services, Decriminalization, Harm reduction

## Abstract

**Introduction:**

Canada and the United States continue to experience increasing overdose deaths attributed to highly toxic illicit substances, driven by fentanyl and its analogues. Many bystanders report being hesitant to call 9-1-1 at an overdose due to fears around police presence and arrests. In Canada, a federal law was enacted in 2017, the *Good Samaritan Drug Overdose Act (GSDOA),* to provide protection from simple drug possession and related charges when 9-1-1 is called to an overdose. There is limited evidence, however, that the *GSDOA* has improved rates of intention to call 9-1-1 at overdose events. We therefore sought to examine intent to call 9-1-1 among persons who received *GSDOA* education and were at risk of witnessing an overdose.

**Methods:**

A cross-sectional survey was conducted with people at risk of witnessing an overdose recruited at 19 Take Home Naloxone (THN) program sites across British Columbia as well as online through Foundry from October 2020 to April 2021. Descriptive statistics were used to examine intention to call 9-1-1 at future overdoses. Multivariable logistic regression models were built in hierarchical fashion to examine factors associated with intention to call 9-1-1.

**Results:**

Overall, 89.6% (*n =* 404) of the eligible sample reported intention to call 9-1-1. In the multivariable model, factors positively associated with intention to call 9-1-1 included identifying as a cisgender woman (adjusted odds ratio [AOR]: 3.37; 95% CI: 1.19–9.50) and having previous *GSDOA* awareness ([AOR]: 4.16; 95% CI: 1.62–10.70). Having experienced a stimulant overdose in the past 6 months was negatively associated with intention to call 9-1-1 ([AOR]: 0.24; 95% CI: 0.09–0.65).

**Conclusion:**

A small proportion of the respondents reported that, despite the enactment of *GSDOA,* they did not intend to call 9-1-1 and those who were aware of the act were more likely to report an intention to call at future overdose events. Increasing *GSDOA* awareness and/or additional interventions to support the aims of the *GSDOA* could address ongoing reluctance to seek emergency medical care by people who use drugs.

**Supplementary Information:**

The online version contains supplementary material available at 10.1186/s13011-022-00484-0.

## Background

Canada and the United States (US) have experienced an increasingly pervasive overdose crisis over recent years [1, 2]. During the 12-month period, ending in April 2021, the US recorded 100,306 overdose deaths [3]. In 2021, Canada also noted a record high in overdose deaths with 5368 apparent opioid toxicity (overdose) deaths occurring between January–September 2021 [4]. The province of British Columbia (BC) has consistently reported the highest rates of illicit drug toxicity (overdose) deaths of any province in Canada since 2016 [1]. Contamination of the illicit drug supply with fentanyl (a synthetic opioid) and its analogues have been key drivers of the crisis [5]. The detected presence of fentanyl among illicit drug toxicity deaths increased from 5% in 2012 to 85% in 2020, which coincides with a more than five-fold increase in illicit drug toxicity deaths over the same period of time [6].

Since BC declared a public health emergency due to illicit drug toxicity deaths in April 2016 [7], a key aspect of the public health response has been to expand the distribution of naloxone, an inhibitor of μ-opioid receptors that can reverse the respiratory depression associated with an opioid-induced overdose [8], through the community-based Take Home Naloxone (THN) program [9]. As of August 2021, there are 1884 active THN distribution sites in BC, which include: harm reduction sites (e.g., harm reduction supply distribution sites and observed consumption sites) [10], hospitals (i.e., all emergency departments), provincial correctional facilities for people on release, community pharmacies, and other sites frequented by people who use substances [11]. These sites provide overdose prevention, recognition, and response training along with low-barrier access to naloxone kits at no cost to individuals at risk of witnessing an overdose. Alongside administering naloxone, THN training emphasizes calling an emergency number, 9-1-1, to alert emergency medical services (EMS) as one of the primary responses to an overdose. Seeking medical services is particularly important, given potent opioids can have a longer duration of effect than naloxone, and there is a risk of reverting back into a state of overdose even after receiving naloxone or experiencing other adverse effects of overdose [12–14].

Despite recommendations to call 9-1-1 when witnessing an overdose, studies in BC have found that bystanders often delay or avoid contacting EMS, with 9-1-1 being called about half of the time [15, 16]. Some of the most commonly reported deterrents to calling 9-1-1 include fears of arrest, general concern of police involvement, previous negative experiences with first responders, and fears around losing housing or custody of children [17–20]. Fears and concerns are especially prominent in youth as studies have found they are more commonly targeted by police compared to adults and are likely to perceive police authority as negative and unpredictable [21–23]. In BC, interventions to reduce these barriers included a regional policy put in place by the Vancouver Police Department in 2006 to not attend overdose calls; this was in response to reported concerns that people did not call 9-1-1 for fear that police would attend. In June 2016, a province wide policy was initiated by BC Emergency Health Services (BCEHS) to not routinely inform police of overdoses except in cases of death, attempted suicide or when there are safety concerns for the public or for first responders [24, 25]. Research conducted in the province suggested that these policies decreased police attendance at overdoses and reduced the proportion of bystanders who reported fears of police presence as a barrier to calling 9-1-1 [15, 25]. However, the non-informing policies did not result in an increased likelihood of bystanders calling 9-1-1 [15]. To further encourage overdose bystanders to contact EMS, the Canadian federal government introduced the *Good Samaritan Drug Overdose Act* (*GSDOA*) in 2017 [26]. Under this legislation, any person at the scene of an overdose, including the person having the overdose, is protected from charges for simple possession of a controlled substance when 9-1-1 is called. Additionally, the *GSDOA* protects people with prior charges and conditions related to simple possession, such as breach of probation. However, the *GSDOA* does not provide legal protection for offences other than simple drug possession, including drug trafficking as well as other offences, such as warrants [26].

The current evidence on the effectiveness of the *GSDOA* and other similar drug related Good Samaritan laws in the US is limited [27]. This has been attributed, in part, to low levels of awareness and understanding of drug related Good Samaritan laws among police officers and people who use drugs (PWUD) [28–31]. Studies comparing overdose mortality across multiple states have had mixed results, where only one of three shows a significant reduction in fatal overdoses in regions where a drug related Good Samaritan law has been enacted [32–34]. Among PWUD and police officers in BC, there is moderate awareness of the *GSDOA*; however, accurate understanding of the Act is low [30, 31, 35]. Even among persons who are aware of their local drug related Good Samaritan law, attitudes and perceptions of its effectiveness are not always positive [19]. A study conducted in BC found that police officers did not have an accurate understanding of the *GSDOA* and reported exercising their discretion to interpret the Act, leading to inconsistencies in its implementation [31]. PWUD also face social and structural barriers to accessing health services, such as stigma and systemic discrimination, which are not accounted for by Good Samaritan laws [36–38].

In 2021, British Columbia’s Office of the Human Rights Commissioner released a report indicating that in BC Indigenous, Black and People of Color are disproportionately subjected to police enforcement practices and violent action from police [39]. It was concluded that systemic racism in police services and other parts of the justice system were a significant driver of these disparities. Systemic discrimination towards Indigenous, Black and People of Color from police officers is not unique to BC. There is a large body of evidence of similar racist and discriminatory policing practices in Canada, more broadly, and the US [40–43]. Indigenous, Black, People of Color who use drugs may be particularly hesitant or unwilling to call 9-1-1 in the event of an emergency due to past experiences and/or concerns around discriminatory responses (e.g. enforcement, substandard medical care, violence) from first responders, and particularly police officers, despite decriminalization under the *GSDOA*. While there exists limited evidence around the impact of Good Samaritan Laws and medical amnesty policies on Indigenous, Black, People of Color’s willingness to call 9-1-1 in the event of a medical emergency, some studies have found that racialized people experience more hesitancy and/or concern due to perceived risks associated with discrimination from first responders [44, 45]. Given these structural vulnerabilities and the *GSDOA's* limited scope, it is likely that some populations who are at risk of witnessing an overdose are not being effectively served by drug related Good Samaritan Laws despite their awareness of the law [35].

Utilizing data from a cross-sectional survey administered across 19 THN program sites in BC, our aim was to investigate factors associated with intention to call 9-1-1 among people who have received education about the *GSDOA*. Our findings can inform targeted initiatives to increase *GSDOA* awareness and address barriers to calling 9-1-1.

## Methods

### Study Design & Data Collection

This study used data from the BC Centre for Disease Control’s (BCCDC) *GSDOA* Survey, administered from October 2020 to April 2021 (See Additional File 1). A cross-sectional survey was designed collaboratively by a team of researchers, regional health authority representatives, harm reduction coordinators, people with lived and living experience (PWLLE) of substance use, youth organizations and youth representatives. The *GSDOA* survey was developed by adapting the annual BC Harm Reduction Client Survey [46–48], using similar demographic and overdose risk and response questions to allow for consistency and potential comparisons. In addition, the *GSDOA* survey included in-depth questions about *GSDOA* awareness and knowledge, experiences with first responders at overdose events and willingness to call 9-1-1 i.e. intention to call 9-1-1 at an overdose event. Survey development was an iterative process; informed by the literature, our research questions and input from adults and youth who were a risk of witnessing an overdose including advisory groups of people with lived and living experience of substance use. The paper and online surveys were piloted to ensure the questions had face validity and response options were clear. Research team members at Foundry, a provincial network of health and social services for youth, provided input throughout the study and members of the Youth4Youth advisory group provided input as youth representatives. Findings were also presented to the Métis Nation of BC as well as peer groups which include the Professionals for Ethical Engagement of Peers (PEEP) and Peer2Peer Project (P2P), and their feedback was incorporated into our analysis and interpretation of findings.

Using input from regional harm reduction coordinators, 19 THN sites with sufficient capacity (staff and physical space accounting for COVID-19 guidelines i.e. physical distancing) from across the province were invited and agreed to participate. In-person survey participants (*n =* 416) were provided $10 CAD and an additional $5 CAD was provided to the participating THN site for each participant enrolled. An online version of the survey was available through Qualtrics [49]. This was offered as an option to respond by THN sites and was advertised by Foundry to recruit youth participants, who were defined as 16–24 years old [50]. Persons completing the survey online (*n =* 77) were offered participation in a raffle for a 1 in 10 chance of obtaining a $50 VISA gift card. Eligibility criteria at THN sites were age (18 years and over) and being at risk of witnessing an overdose. This included PWLLE, peer responders and family or friends of people who use drugs as these individuals have a higher likelihood of witnessing an overdose [51–53]. Institutional ethics approval was obtained through the University of British Columbia’s Behavioural Research Ethics Board (# H19–01842).

### Study variables

#### Outcome variable

**Fig. 1:**
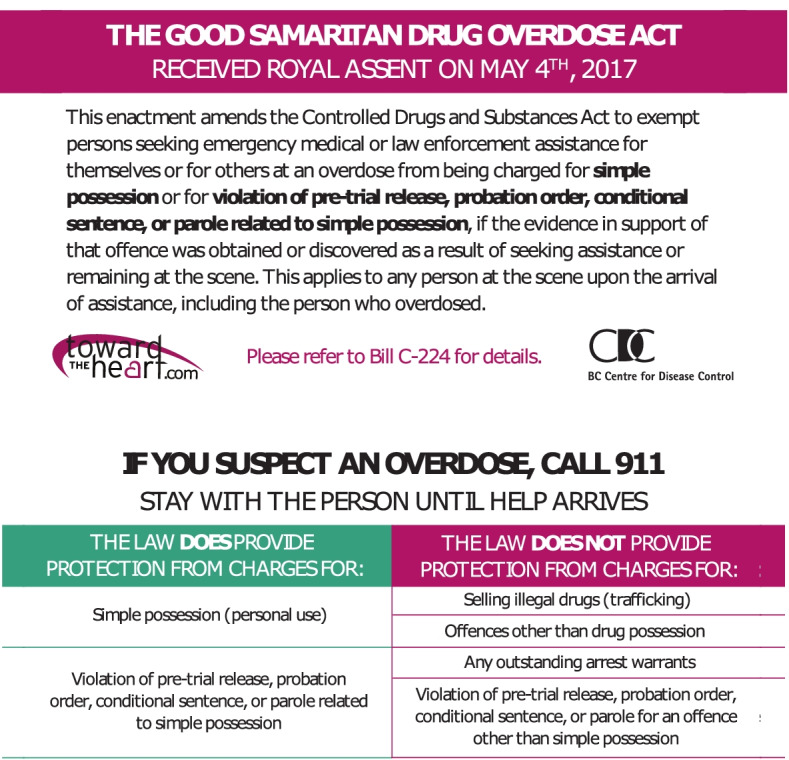
Wallet cards outlining the tenets of the GSDOA that are distributed in the community

The primary outcome variable for this study was “Intention to call 9-1-1 at an overdose event”. After being provided with a definition of the *GSDOA* (Fig. 1), participants were asked “*Based on this description, if you were to witness someone overdose in the future, would you call 9-1-1?*” to which they could answer one of “yes”, “no” or “prefer not to say”. Included in the question was the note “*Disclaimer: We cannot guarantee that police and emergency health responders will be knowledgeable about the GSDOA and will follow the Act.*”

#### Explanatory variables

Explanatory variables of interest included participants’ sociodemographic and substance use characteristics as well as variables that reflected participants’ *GSDOA* knowledge and awareness. Sociodemographic characteristics included age group (16–24, 25–34, 35–54, ≥55 years), gender (cisgender men, cisgender women, transgender and gender expansive [trans-men, trans-women, gender non-binary], prefer not to say), Indigenous identity (non-Indigenous, Indigenous [First Nations, Métis, Inuit], prefer not to say), geographic health region as defined by the five health authorities within the province (Fraser Health, Interior Health, Island Health, Northern Health, Vancouver Coastal Health), employment status (yes, no, prefer not to say), housing status (private alone, private with other(s), supportive or unstable housing [hotel, motel, rooming house, single room occupancy, shelter], homeless, prefer not to say) and cellphone possession (yes, no, prefer not to say). Due to a small number of participants reporting gender identity as “trans and gender expansive”, we excluded this group and used a binary gender identity variable of “cis man” and “cis woman” for statistical analyses, though they were retained for descriptive analysis (Table 1).

Indigenous identity is understood to act as a proxy for factors associated with colonialism including intergenerational trauma, systemic racism, criminalization and discrimination [54–57]. Descriptive analyses of First Nations, Métis, Inuit, and non-Indigenous identity were included in recognition of the heterogeneity of Indigenous peoples and their experiences (Table 1). However, Indigenous identity was dichotomized to maintain sample size in statistical analysis and regression models (Tables 2 and 3).

Prior awareness of the *GSDOA* was determined by asking participants if they were aware of the *GSDOA* before the definition of the Act was provided. To evaluate understanding of the *GSDOA*, a set of questions were included that had been used previously in Mehta et al. [35]. Briefly, hypothetical overdose scenarios were outlined and participants were asked true or false questions to assess their knowledge of *when* and *to whom* protection is offered under the *GSDOA*. Knowledge was considered “complete” if all questions were answered correctly and “incomplete” if otherwise (See Additional File 1).

To assess perceived risks of experiencing or witnessing an overdose, respondents were asked to rate the degree to which they felt at risk of these events in the previous 6 months using a Likert-type scale. Possible responses were “never”, “rarely”, “sometimes”, “often” or “all the time” which was dichotomized as “never” and “ever”. Additional collected variables on substance use and overdose experience in the last 6 months included using opioids (yes, no, prefer not to say), overdosing on opioids (yes, no, don’t know, prefer not to say), witnessing an opioid overdose (yes, no, don’t know, prefer not to say), overdosing on stimulants (yes, no, don’t know, prefer not to say) and witnessing a stimulant overdose (yes, no, don’t know, prefer not to say).

### Data analysis

All analyses were conducted using R version 4.0.2 [58]. Frequency distributions and bivariate analyses with chi-square tests of independence were conducted to describe characteristics of participants and to explore relationships between intention to call 9-1-1 and the explanatory variables.

For multivariable analysis, candidate variables were separated into relevant categories, or blocks, that were organized by linking conceptual similarities through a concept map (See Supplementary Fig. 1, Additional File 2). Notably, Indigeneity was separated from other demographic characteristics because it acts as a proxy for a number of other factors relating to colonialism as described above. Hierarchical logistic regression was then used to estimate the association of these blocks and their variables with intention to call 9-1-1 [59]. The final model was entered block by block in five steps:

1. Demographic characteristics except for Indigeneity (age, gender, health region)
2. Indigeneity (identifying as First Nations, Métis and/or Inuit)
3. Socioeconomic status characteristics (housing status, employment status)
4. Overdose response resources (cellphone possession, prior *GSDOA* awareness, complete understanding of the *GSDOA*)
5. Overdose characteristics (perceived risk of overdose, perceived risk of witnessing an overdose, stimulant overdose experienced, stimulant overdose witnessed, opioid overdose experienced, opioid overdose witnessed)

To build the model within each block, bivariate logistic regression of each explanatory variable with the outcome variable was completed and variables with *p* value < 0.25 were considered for selection [60, 61]. Variables were then selected through a backwards selection approach based on minimizing the value of Akaike’s information criteria (AIC) [62]. Conceptually important variables were retained in the model (i.e. age). The final selected model included: age, gender, Indigenous identity, housing status, employment status, previous awareness of the *GSDOA* and having experienced a stimulant overdose in the past 6 months. To illustrate the relative contribution of each block to model fit, likelihood ratio R^2^ was calculated after incorporating each additional block [63]. Models were also compared using the Likelihood Ratio Test with each nested model being compared to the model generated in the previous step [60]. The theory of planned behaviour was used as a conceptual framework to inform interpretation and discussion of results [64]. Briefly, the theory of planned behaviour posits that attitudes toward a behaviour, subjective norms and perceived behavioural controls influence the formation of a behavioral intention and this can be used to predict or understand the context of certain behaviours which, in our current study, is calling 9-1-1 for an overdose.

### Missing data

Complete case analysis (CCA) was used for this study. This resulted in the exclusion of individuals with missing, “prefer not to say” or “don’t know” responses which were categorized as “unknown” (Fig.﻿ 2). In total, 153 (33.7%) observations were removed from the analysis and a total of 327 responses were used for the multivariable model. The model was rerun in sensitivity analyses with a dataset where all unknown values were imputed with multiple imputation by chained equation (MICE) [65]. Briefly, a parallel analysis was conducted using ten imputed datasets each generated by ten cycles of MICE [66]. Results were verified by comparing the imputed model to the unimputed model and there were no significant differences in the conclusions, confirming confidence in the results of the CCA.

## Results

### Demographic characteristics of study participants

**Fig. 2:**
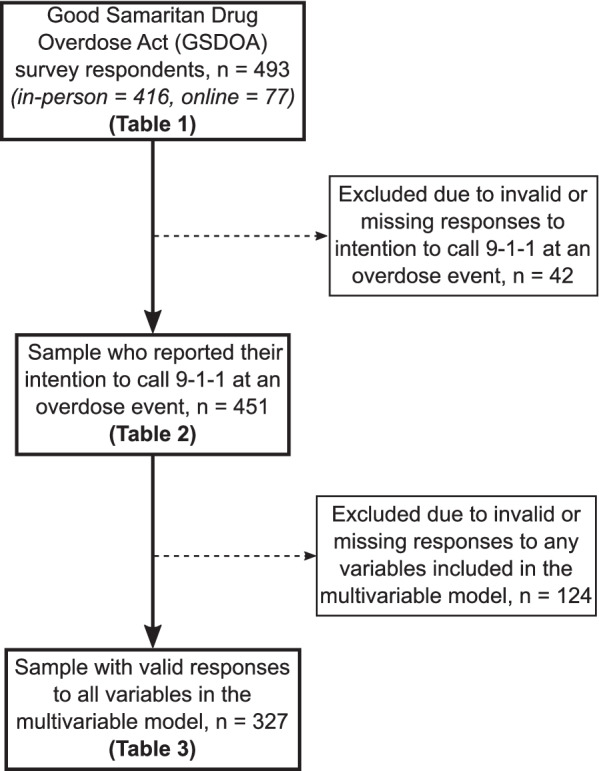
Outline of the study sample used for each analysis

A total of 493 participants completed the *GSDOA* survey (Fig. 2). Demographic characteristics are presented in Table 1 stratified by health region. Participants were evenly distributed across health regions except for a smaller proportion from the Northern Health region (9.3%) due to population rurality and remoteness. Almost a quarter (23.4%) of participants who reported their age were in the youth age group (16–24 years), 61.8% were between 25 and 54 years old, and 14.7% were 55 years and over. Among participants who reported gender identity, the majority were cis men (57.7%) followed by cis women (37.6%) and trans and gender expansive people (4.7%). Of those who responded to the question on Indigenous identity, a total of 55.8% identified as non-Indigenous, 29.7% identified as First Nations, 13.8% as Métis and 0.7% as Inuit.

**Table 1:** Demographics of the *GSDOA* Survey (2020–2021) by the BC health regions (*N =* 493)

Demographic Characteristics	Fraser Health *n* (row %)	Interior Health *n* (row %)	Island Health *n* (row %)	Northern Health *n* (row %)	Vancouver Coastal Health *n* (row %)	Total *N* (column %)
**Participants**	104 (21.1)	128 (26.0)	100 (20.3)	46 (9.3)	115 (23.3)	493 (100)
**Age (years)**						
16–24 years	16 (14.2)	14 (12.4)	17 (15.0)	9 (8.0)	57 (50.4)	113 (22.9)
25–34 years	22 (23.7)	25 (26.9)	24 (25.8)	9 (9.7)	13 (14.0)	93 (18.9)
35–44 years	25 (23.4)	36 (33.6)	23 (21.5)	10 (9.3)	13 (12.1)	107 (21.7)
45–54 years	17 (17.3)	30 (30.6)	24 (24.5)	12 (12.2)	15 (15.3)	98 (19.9)
55 years and over	20 (28.2)	20 (28.2)	11 (15.5)	5 (7.0)	15 (21.1)	71 (14.4)
*Unknown*	4 (36.4)	3 (27.3)	1 (9.1)	1 (9.1)	2 (18.2)	11 (2.2)
**Gender identity**						
Cis man	62 (22.0)	79 (28.0)	61 (21.6)	21 (7.4)	59 (20.9)	282 (57.2)
Cis woman	37 (20.1)	46 (25.0)	35 (19.0)	24 (13.0)	42 (22.8)	184 (37.3)
Trans and gender expansive ^a^	4 (17.4)	3 (13.0)	3 (13.0)	1 (4.3)	12 (52.2)	23 (4.7)
*Unknown*	1 (25.0)	0 (0.0)	1 (25.0)	0 (0.0)	2 (50.0)	4 (0.8)
**Indigenous identity**^**b**^						
First Nations	25 (18.8)	30 (22.6)	19 (14.3)	20 (15.0)	39 (29.3)	133 (27.0)
Métis	12 (19.4)	21 (33.9)	11 (17.7)	13 (21.0)	5 (8.1)	62 (12.6)
Inuit	2 (66.7)	0 (0.0)	1 (33.3)	0 (0.0)	0 (0.0)	3 (0.6)
Non-indigenous	54 (21.4)	60 (23.8)	63 (25.0)	10 (4.0)	65 (25.8)	252 (51.1)
*Unknown*	11 (25.6)	17 (39.5)	6 (14.0)	3 (7.0)	6 (14.0)	43 (8.7)

Health regions in increasing order of population are: Northern Health, Interior Health, Island Health, Vancouver Coastal Health, Fraser Health.

^a^ “Trans and gender expansive” identities include trans man, trans woman and gender non-conforming people

^b^ The authors recognize that Indigenous identity is often a proxy for factors associated with colonialism including intergenerational trauma, systemic racism, criminalization and discrimination

Table 2 presents the distribution of the study variables stratified by intention to call 9-1-1 at an overdose event (*n =* 451) with most (89.6%) reporting that they would call 9-1-1. Youth (16–24 years) made up the largest group in this sample (23.7%) and the majority identified as cis men (57.0%). Furthermore, a large portion of this sample identified as non-Indigenous (52.1%), lived in supportive/unstable housing (46.1%), were unemployed (63.6%) and owned a cellphone (65.0%). Approximately half (49.0%) of the respondents had previously heard of the *GSDOA* and, of those, about half (50.2%) had complete knowledge of who is protected from charges for simple possession of a controlled substance under the *GSDOA*. Of those who were aware of the Act, a third (33.9%) had complete knowledge of when protection is offered under the *GSDOA*. Over the prior 6 months, 50.8% of respondents felt at some risk of experiencing an overdose and 85.1% felt at some risk of witnessing an overdose. In the last 6 months, over half reported using opioids (55.7%), 18.0% had experienced an opioid overdose, and 15.3% had experienced a stimulant overdose. The majority of participants had witnessed an opioid overdose (57.2%).

**Table 2:** Factors associated with intention to call 9-1-1 at a future overdose among survey respondents

	Intention to call 9-1-1 at overdose events			
	Yes (*N =* 404) *n* (row %)	No (*N =* 47) *n* (row %)	Total (*N =* 451) *n* (column %)	***P-***value ^a^
**Age (years)**				0.126
16–24 years	101 (94.4)	6 (5.6)	107 (23.7)	
25–34 years	70 (85.4)	12 (14.6)	82 (18.2)	
35–44 years	86 (87.8)	12 (12.2)	98 (21.7)	
45–54 years	87 (94.6)	5 (5.4)	92 (20.4)	
55 years and over	57 (89.1)	7 (10.9)	64 (14.2)	
*Unknown*	3 (37.5)	5 (62.5)	8 (1.8)	
**Gender identity** ^b^				0.091
Cis man	225 (87.5)	32 (12.5)	257 (57.0)	
Cis woman	159 (93.0)	12 (7.0)	171 (37.9)	
Trans and gender expansive	20 (90.9)	2 (9.1)	22 (4.9)	
*Unknown*	0 (0.0)	1 (100.0)	1 (0.2)	
**Indigenous identity**^**c**^				0.164
Indigenous	150 (87.2)	22 (12.8)	172 (38.1)	
Non-Indigenous	216 (91.9)	19 (8.1)	235 (52.1)	
*Unknown*	38 (86.4)	6 (13.6)	44 (9.8)	
**Health region**				0.683
Fraser	86 (89.6)	10 (10.4)	96 (21.3)	
Interior	106 (89.1)	13 (10.9)	119 (26.4)	
Island	81 (86.2)	13 (13.8)	94 (20.8)	
Northern	36 (92.3)	3 (7.7)	39 (8.6)	
Vancouver Coastal	95 (92.2)	8 (7.8)	103 (22.8)	
**Housing status**				**< 0.01**
Private/Alone	40 (71.4)	16 (28.6)	56 (12.4)	
Private/With Other(s)	107 (93.0)	8 (7.0)	115 (25.5)	
Supportive/Unstable Housing	193 (92.8)	15 (7.2)	208 (46.1)	
Homeless	53 (89.8)	6 (10.2)	59 (13.1)	
*Unknown*	11 (84.6)	2 (15.4)	13 (2.9)	
**Employment**				**0.029**
Yes	126 (85.1)	22 (14.9)	148 (32.8)	
No	264 (92.0)	23 (8.0)	287 (63.6)	
*Unknown*	14 (87.5)	2 (12.5)	16 (3.5)	
**Cellphone possession**				**0.027**
Yes	269 (91.8)	24 (8.2)	293 (65.0)	
No	118 (84.3)	22 (15.7)	140 (31.0)	
*Unknown*	17 (94.4)	1 (5.6)	18 (4.0)	
**Previous** ***GSDOA*** **awareness**				**0.019**
Yes	205 (92.8)	16 (7.2)	221 (49.0)	
No	173 (85.2)	30 (14.8)	203 (45.0)	
*Unknown*	26 (96.3)	1 (3.7)	27 (6.0)	
**Complete knowledge of whom the** ***GSDOA*** **protects** ^d^				0.116
Yes	106 (95.5)	5 (4.5)	111 (24.6)	
No	99 (90.0)	11 (10.0)	110 (24.4)	
Unaware	173 (85.2)	30 (14.8)	203 (45.0)	
*Unknown*	26 (96.3)	1 (3.7)	27 (6.0)	
**Complete knowledge of when the** ***GSDOA*** **protects** ^e^				0.646
Yes	71 (94.7)	4 (5.3)	75 (16.6)	
No	134 (91.8)	12 (8.2)	146 (32.4)	
Unaware	173 (85.2)	30 (14.8)	203 (45.0)	
*Unknown*	26 (96.3)	1 (3.7)	27 (6.0)	
**Perceived risk of experiencing an overdose** (last 6 months)				0.721
Never	192 (90.6)	20 (9.4)	212 (47.0)	
Ever	204 (89.1)	25 (10.9)	229 (50.8)	
*Unknown*	8 (80.0)	2 (20.0)	10 (2.2)	
**Perceived risk of witnessing an overdose** (last 6 months)				0.292
Never	45 (84.9)	8 (15.1)	53 (11.8)	
Ever	348 (90.6)	36 (9.4)	384 (85.1)	
*Unknown*	11 (78.6)	3 (21.4)	14 (3.1)	
**Opioid use** (last 6 months)				0.999
Yes	224 (89.2)	27 (10.8)	251 (55.7)	
No	141 (89.8)	16 (10.2)	157 (34.8)	
*Unknown*	39 (90.7)	4 (9.3)	43 (9.5)	
**Opioid overdose** (last 6 months) ^e^				0.854
Yes	73 (90.1)	8 (9.9)	81 (18.0)	
No	307 (90.8)	31 (9.2)	338 (74.9)	
*Unknown*	24 (75.0)	8 (25.0)	32 (7.1)	
**Stimulant overdose** (last 6 months)				0.066
Yes	58 (84.1)	11 (15.9)	69 (15.3)	
No	321 (92.0)	28 (8.0)	349 (77.4)	
*Unknown*	25 (75.8)	8 (24.2)	33 (7.3)	
**Opioid overdose witnessed** (last 6 months)				0.611
Yes	233 (90.3)	25 (9.7)	258 (57.2)	
No	133 (92.4)	11 (7.6)	144 (31.9)	
*Unknown*	38 (77.6)	11 (22.4)	49 (10.9)	
**Stimulant overdose witnessed** (last 6 months)				0.675
Yes	159 (90.9)	16 (9.1)	175 (38.8)	
No	204 (89.1)	25 (10.9)	229 (50.8)	
*Unknown*	41 (87.2)	6 (12.8)	47 (10.4)	

^a^ Chi square test exclude participants with unknown independent variables

^b^ “Trans and gender expansive” is shown but is not included in the chi square test due to small sample size

^c^ The authors recognize that Indigenous identity is often a proxy for factors associated with colonialism including intergenerational trauma, systemic racism, criminalization and discrimination

^d^ “Unaware” is shown but is not included in the chi square test

^e^ “Didn’t use opioids” is shown but is not included in the chi square test

Willingness to call 9-1-1 was reported by a higher proportion of participants who were unemployed (92.0%) compared to employed (85.1%), participants who owned a cellphone (91.8%) compared to those who did not (84.3%), and participants who were previously aware of the *GSDOA* (92.8%) compared to those who were unaware (85.2%). Willingness to call 9-1-1 was greater among respondents living in private housing with others (93.0%), living in supportive/unstable housing (92.8%) and persons experiencing homelessness (89.8%) compared to respondents who lived alone in private housing (71.4%) (all *p* < 0.05).

### Factors associated with an intention to call 9-1-1 at an overdose event

Unadjusted bivariate and adjusted odds ratios from multivariable models that estimate the likelihood of intention to call 9-1-1 at an overdose event are shown in Table 3. Age was considered conceptually important and was therefore retained in the model. Models were constructed hierarchically to assess the influence of demographic characteristics, Indigeneity, socioeconomic status (SES) factors, overdose response resources and overdose characteristics on intention to call 9-1-1 at an overdose event. Demographic characteristics were not significantly associated with intention to call 9-1-1 (χ^2^ = 10.25, *p* = 0.68). Nevertheless, cis women were found to have more than twice the odds of intending to call 9-1-1 for an overdose compared to cis men (AOR = 2.59 [95% CI 1.01, 6.68]) after adjusting for age. The addition of Indigeneity significantly improved the model fit (χ^2^ = 5.58, *p* = 0.018) and persons who identified as Indigenous had significantly lower odds of intending to call 9-1-1 in the second hierarchical model (AOR = 0.38 [95% CI 0.17, 0.85]) after adjusting for age and gender. Further adding SES factors did not significantly improve the model fit in terms of predicting intention to call 9-1-1 (χ^2^ = 7.74, *p* = 0.102). However, results indicated that respondents in supportive/unstable housing (AOR = 3.40 [95% CI 1.18, 9.78]) and persons living with others in private housing (AOR = 3.90 [95% CI 1.01, 15.03]) had elevated odds of being willing to call 9-1-1 at an overdose event after adjusting for age, gender and Indigeneity. The addition of overdose response resources further improved model fit (χ^2^ = 8.20, *p* < 0.01). Persons who reported prior awareness of the *GSDOA* had over three time the odds of being willing to call 9-1-1 (AOR = 3.52 [95% CI 1.42, 8.69]) after adjusting for age, gender, Indigeneity, housing status and employment status. Including overdose characteristics significantly improved the fit of the model (χ^2^ = 7.60, *p* < 0.01). Participants who had experienced a stimulant overdose in the past 6 months had significantly lower odds of intention to call 9-1-1 at an overdose event (AOR = 0.24 [95% CI 0.09, 0.65]) after adjusting for age, gender, Indigeneity, housing status, employment status and previous *GSDOA* awareness.

**Table 3:** Estimated odds ratios (OR) and adjusted odds ratios (AOR) for predictors of intention to call 911 at a future overdose among participants

	Calling 911 at an OD event^a^					
Variables	Bivariate OR (95% CI)	Block 1 (Demographics) AOR (95% CI)	Block 2 (Indigenous Identity) AOR (95% CI)	Block 3 (SES) AOR (95% CI)	Block 4 (OD Response) AOR (95% CI)	Block 5 (OD Characteristics) AOR (95% CI)
**Demographic Characteristics**						
*Age (years)*						
16–24	–	–	–	–	–	–
25–34	0.35 (0.09, 1.40)	0.41 (0.10, 1.70)	0.40 (0.10, 1.66)	0.35 (0.08, 1.57)	0.28 (0.06, 1.31)	0.20 (0.04, 0.98) *
35–44	0.34 (0.09, 1.35)	0.43 (0.11, 1.74)	0.41 (0.10, 1.65)	0.34 (0.08, 1.52)	0.32 (0.07, 1.47)	0.24 (0.05, 1.18)
45–54	0.71 (0.15, 3.28)	0.88 (0.19, 4.13)	0.91 (0.19, 4.30)	0.81 (0.16, 4.12)	0.81 (0.15, 4.29)	0.55 (0.10, 3.07)
55 +	0.24 (0.06, 1.03)	0.30 (0.07, 1.32)	0.26 (0.06, 1.13)	0.25 (0.05, 1.18)	0.25 (0.05, 1.25)	0.17 (0.03, 0.90) *
*Gender*						
Cis man	–	–	–	–	–	–
Cis woman	2.90 (1.15, 7.35) *	2.59 (1.01, 6.68) *	3.00 (1.15, 7.83) *	3.05 (1.14, 8.15) *	3.24 (1.19, 8.84) *	3.37 (1.19, 9.50) *
**Indigenous Identity**^**b**^						
Non-indigenous	–		–	–	–	–
Indigenous	0.46 (0.21, 1.01)		0.38 (0.17, 0.85) *	0.43 (0.18, 1.00)	0.48 (0.20, 1.15)	0.64 (0.25, 1.64)
**SES Factors**						
*Housing Status*						
Private - Alone	–			–	–	–
Private - With others	4.85 (1.37, 17.21) *			3.90 (1.01, 15.03) *	3.65 (0.94, 14.26)	4.96 (1.21, 20.29) *
Supportive/Unstable Housing	3.17 (1.18, 8.51) *			3.40 (1.18, 9.78) *	3.56 (1.20, 10.64) *	3.96 (1.31, 11.98) *
Homeless	2.04 (0.61, 6.81)			1.91 (0.51, 7.16)	2.08 (0.53, 8.16)	2.39 (0.58, 9.82)
*Employment Status*						
Not employed	–			–	–	–
Employed	0.65 (0.30, 1.43)			0.52 (0.21, 1.26)	0.51 (0.20, 1.28)	0.46 (0.18, 1.18)
**Overdose Response Resources**						
*Previous awareness of GSDOA*						
Unaware	–				–	–
Aware	3.16 (1.35, 7.41) **				3.52 (1.42, 8.69) **	4.16 (1.62, 10.7) **
**Overdose Characteristics**						
*Stimulant OD*						
No	–					–
Yes	0.36 (0.15, 0.81) *					0.24 (0.09, 0.65) **
*LR Pseudo–R*^*2*^		0.054	0.083	0.123	0.166	0.206
*Pseudo–R*^*2*^ *change*		0.054	0.029 *	0.040	0.043 **	0.040 **

Note: *SES* Socioeconomic status, *OD* Overdose. Reference categories are denoted by —. *p < 0.05, **p < 0.01

^a^ Final model size *N =* 327 after excluding individuals with “unknown” responses for all variables

^b^ The authors recognize that Indigenous identity is often a proxy for factors associated with colonialism including intergenerational trauma, systemic racism, criminalization and discrimination

To address missing data, a multivariable model was built containing the same variables but using pooled imputed datasets generated by multiple imputation by chained equation (*N =* 493) (See Supplementary Table 1, Additional File 2). Though the strength of association changed for some explanatory variables, the directions of the associations of interest were consistent with unimputed results from Table 3.

## Discussion

About 10% of the sample reported that the *GSDOA* did not affect their intent to call 9-1-1 at a future overdose event and a number of characteristics have been found to be associated with this intent. We found higher odds of intention to call 9-1-1 among cis women, non-Indigenous respondents, participants living in supportive, unstable or private housing with others compared to private housing alone, persons with prior awareness of the *GSDOA*, and participants who had not experienced a stimulant overdose over the last 6 months. Importantly, demographic factors, apart from Indigeneity, and SES factors did not significantly improve the model fit. Overdose response resources and overdose characteristics each accounted for a significant portion of the variation in the outcome variable over and above the factors included before them. This suggests that, in our model, demographic and SES factors had a relatively less important role in predicting intention to call 9-1-1 at an overdose compared to Indigeneity and overdose-related factors.

Previous studies have assessed factors associated with calling 9-1-1 at an overdose among samples of people with variable knowledge of drug related Good Samaritan laws, which have been implemented in many states throughout the US [15–17, 67, 68]. Although our study included a substantial proportion of youth (aged 16–24), a population that has previously been identified as reticent to call 9-1-1 due to fear of criminalization and mistrust of police [6, 23, 69, 70], we did not find that intent to call 9-1-1 differed by this age group (Block 1). Future research should investigate the potential age-related dynamics to intention to call 9-1-1 at overdose events given prior research in this area. In addition, we found that cis women were more likely to express intention to call 9-1-1 at a future overdose event. This broadly corroborates previous research on sex and gender that found a higher likelihood of 9-1-1 being called when female bystanders were present [16, 17]. Critical feminist scholars suggest that gender is socially prescribed and devised largely based on social conditioning ([71], cited by [72], cited by [73]). Consequently, a body of literature argues that the high prevalence of women in caregiving roles is a product of processes of socialization, and associated service landscapes and policies ([74–76], cited by [72], [77], cited by [73]). As studies by Brewer [73] and Doyal [78] suggest, power relations and societal influences are likely at the root of the burden of unpaid caregiving work disproportionately falling on women. Furthermore, norms surrounding masculinity that reject vulnerability and contribute to lower medical care seeking may contribute to reduced odds of calling 911 among cis-men [79, 80]. Studies examining THN program uptake have revealed that structural power and social dynamics such as these are central to overdose response landscapes [81–85]. To be successful, harm reduction programs and policy involving peer action must be sensitive to the social environment of the responder. Taken together with our findings, further research is necessary to elucidate the role of gender on seeking help at overdose events.

We also found decreased likelihood of calling 9-1-1 among people who have overdosed on stimulants in the last 6 months. Ambrose et al. [16] similarly found that the odds of calling 9-1-1 are lower if persons had a previous stimulant overdose. The effects of using stimulants are different from those of opioids, and may impact help seeking and interactions with first responders who can have stigmatizing perceptions of people who use stimulants [86, 87]. Consequently, compared to those who use opioids, people who use stimulants have increased odds of being incarcerated and are more likely to experience an overnight hospital stay [88, 89]. Therefore, people who use stimulants may experience additional risk of harm at overdose events, which may affect attitudes around calling 9-1-1 at future overdose events. More research is needed to confirm this. In addition, our findings could also be reflective of a lower perceived need for naloxone, calling 9-1-1 and the *GSDOA* among people who use stimulants compared to people who use only opioids. A recent study conducted in Vancouver, BC by Mansoor et al. [90] found that, relative to our understanding of the signs of an opioid overdose, a unified understanding of the signs of a stimulant overdose may be lacking despite severe outcomes that can result such as cardiac arrest and seizure. As this study suggests, stimulant overdose recognition and response may be impacted contributing to a higher likelihood of not calling 9-1-1 and self-managing stimulant overdoses [90].

Our finding that those living in a private residence alone had lower odds of intention to call 9-1-1 compared to persons living in private housing with others and persons in supportive or unstable housing is supported by past research. Specifically, a study in New York City, US and two studies conducted in BC found decreased odds of calling 9-1-1 for overdoses occurring in private residences and a higher likelihood of 9-1-1 being called if an overdose was witnessed in a public setting (e.g. on the street) [15, 16, 28]. Additionally, a recent Vancouver study found lower odds of calling 9-1-1 among persons living in SROs compared to other private residences [68]. Bystanders to overdose in private residences may be concerned about lacking anonymity or losing housing as a result of drug use being discovered [91, 92]. This could be particularly relevant for people who live alone as the presence of emergency personnel at their residence could be directly linked to them as would potential repercussions such as loss of housing or eviction.

Respondents who were previously aware of the *GSDOA* had a higher likelihood of intention to call 9-1-1 at future overdose events. Past research has provided evidence for an association between awareness of the *GSDOA* and intent to call 9-1-1 [28, 67, 93–95]. Since all respondents were educated about the *GSDOA*, our finding suggests that reinforcing education of the Act can increase intention to call 9-1-1. Jakubowski et al. [28] had similar results with people who received repeated Good Samaritan law instruction at 3-, 6- and 12-month follow-up points and saw correct knowledge of the Good Samaritan law increase each time. This reinforces the importance of expanding *GSDOA* awareness and knowledge as well as continuing targeted dissemination of the Act.

Our findings indicated that participants who identified as Indigenous had lower odds of intending to call 9-1-1. The addition of subsequent blocks to the hierarchical model reduced this effect to the point where it lacked statistical significance, suggesting that there may have been other factors contributing to this association. Indigenous peoples are racially profiled and mistreated by police and health care professionals in Canada [96–98]. Compared to non-Indigenous peoples, Indigenous peoples are disproportinately incarcerated, making up over 25% of the correctional facility population while only representing 4.5% of the Canadian population [99, 100]. This is largely a result of the ongoing impacts of colonization, including anti-Indigenous discrimination. Indigenous peoples contend with precarious encounters with law enforcement, characterized by risks of violence and discrimination, which contribute to a fear or reduced willingness to contact and interact with the police [101]. Our study may reflect these barriers to calling 9-1-1 among Indigenous respondents that are not addressed by the *GSDOA* [54–57]. The 2020 report *In Plain Sight: Addressing Indigenous-Specific Racism and Discrimination in B.C. Health Care* contains policy recommendations meant to address interpersonal and institutional anti-Indigenous racism in health setting [102]. This includes developing an *Anti-Racism Act* and amending the *Health Professions Act*, *Hospitals Act*, and *Health Authorities Act* to set standards and expectations of anti-racism and Indigenous cultural safety for employees in the health care system including policymakers, clinicians and paramedics. The *GSDOA* cannot be separated from the sociopolitical context in which it operates as forces such as anti-Indigenous racism certainly have an impact on how PWUD experience the *GSDOA* as well as their interactions with first responders and hospital personnel. Policies addressing racism and promoting equitable access to EMS are paramount for the effectiveness of policies such as the *GSDOA*. To further improve implementation of the *GSDOA*, efforts should focus on constant and reflective relationship-building efforts between law enforcement and populations who may be distrustful of police.

To better connect our results to behaviour intentions and identify areas that may respond to interventions, we drew on the theory of planned behaviour as a conceptual framework [64]. According to the theory of planned behaviour, attitudes toward a behaviour, subjective norms and perceived behavioural controls combine to form a behavioral intention (i.e., intention to call 9-1-1 at an overdose event). Indigenous, Black and People of Colour have and continue to report negative interactions with police officers and this has led to attitudes of distrust towards police among these groups [39, 96–98]. Furthermore, people experiencing homelessness and PWUD continue to be criminalized by police [19, 23]. This may be compounded by the subjective norm that one should not call or involve police as this may be perceived as “ratting” on others and potentially subject others to harms associated with police involvement [19, 103]. Masculinity is another subjective norm to consider as this has been shown to lower odds of calling 9-1-1 in men [79, 80]. Though none of the associated factors in our model can be readily described as a perceived behavioural control, one important control to consider from outside the model is cellphone possession. Specifically, over a third of respondents did not have a cellphone and therefore had a potential added barrier to calling 9-1-1 at future overdose events. Furthermore, willingness to call 9-1-1 differed significantly between cellphone owners and those who did not own a cellphone. Altogether, the application of the theory of planned behaviour to our findings highlights that one’s intention to call 9-1-1 at overdose events is complicated by many overlapping influences that operate on both the individual- and environmental-level. Additional qualitative and quantitative research is required to capture the many unique scenarios and circumstances that impact overdose bystanders’ decision-making process.

Despite the sample indicating a high level of intent to call after receiving education about the *GSDOA*, about 10 % of the sample indicated they would not call. The disparities in intention to call 9-1-1 highlight the need for several interventions to support the aims of the *GSDOA*. First, the protections of the *GSDOA* should be expanded to protect bystanders who may fear losing housing or employment if they call 9-1-1. Many PWUD are engaged in criminalized work (e.g. sex work, drug dealing) and this should be taken into account when expanding *GSDOA* protections [104, 105]. Further, instances of police discretion should be minimized to limit inconsistencies in how people at overdose events experience the *GSDOA* [31, 106]. *De jure* decriminalization policies that are developed in collaboration with PWLLE are also needed to address the limitations of the narrow form of decriminalization that is the *GSDOA*. Calls and recommendations from the *In Plain* Sight report [102] should be followed to improve access to care and interactions with first responders for Indigenous, Black and People of Colour. Considering the studies in BC that have highlighted low to moderate levels of *GSDOA* awareness and understanding among PWUD and police officers [30, 31, 35], continued knowledge dissemination efforts, especially to subgroups with lower *GSDOA* awareness and understanding, is warranted and necessary to improve the implementation of the *GSDOA.*

Our study identifies a number of gaps in the literature and potential future research directions. More research is needed to clarify the impact of gender on intent to call 9-1-1 at overdose events, through a structural sexism lens. We did not find that intent to call 9-1-1 was associated with age, however, there are mixed findings in the literature – suggesting that more research is needed. Future research would additionally benefit from exploring experiences at stimulant overdose events, including perspectives and concerns around identifying a stimulant overdose, responding and calling 9-1-1. Lastly, given the multitude of factors, beyond *GSDOA* awareness and knowledge and variables discussed in our analysis, that can contribute to decision-making around help seeking, quantitative and qualitative studies focusing on various and potentially intersecting facilitators and barriers to calling 9-1-1 are needed.

The findings of this study have several limitations. Respondents were recruited by convenience sampling and this research relied largely on respondents at THN sites. Although the study used THN and Foundry sites in all the health regions of BC, most sites were in urban locations and may not therefore adequately capture the perceptions and experiences of people residing in rural areas. The data we collected and analyzed is not all-inclusive and there may be factors that are associated with intention to call 9-1-1 at overdose events that we did not account for (e.g. past negative experiences with first reponders and/or healthcare providers). We did not assess level of education which may have been associated with the outcome – possibly as a result of differences in access to information and learning abilities affecting awareness and correct knowledge surrounding the *GSDOA*. Indigenous identity was assessed, however race and ethnicity was not assessed for those who identified as non-Indigenous. Future studies should assess potential differences in willingness to call 9-1-1 and interactions with police between Indigenous, Black and People of Color and non-racialized people. Though the *GSDOA* has been implemented on the federal level in Canada, this study exclusively took place in BC and may not be applicable to other provinces or countries with similar drug related Good Samaritan laws, such as the USA. These limitations altogether impact the generalizability of the study to all people who may witness an overdose. Additionally, we cannot determine the temporality between the explanatory and outcome variables due to the cross-sectional design of the study. Due to the self-reported nature of our survey, we also cannot rule out certain respondent biases (e.g. confirmation bias, recall bias, social desirability bias). Altogether, the level of intent to call 9-1-1 may be overestimated, especially considering that most participants were recruited at THN sites and those with THN training and experience administering naloxone are more likely to call 9-1-1 for an overdose [68]. Sensitivity analysis was completed using imputed datasets and, while the direction of the main associations of interest remained unchanged, associations of the outcome variable with other covariates differed. This limits our ability to make conclusions about the magnitude of the relationships given that they differ across the imputed and unimputed models.

## Conclusions

This study uniquely assessed the level of intent and factors associated with intent to call 9-1-1 at future overdose events across 19 THN program sites in BC among people at risk of witnessing an overdose and who were educated about the *GSDOA*. Our findings show that about 10% of the sample indicated that the *GSDOA* did not affect their intent to call 9-1-1 at a future overdose event. Our recommendations, informed by the theory of planned behaviour, include public education of the *GSDOA*, the expansion of the *GSDOA’s* legal protections*,* implementation of broader decriminalization and the development of anti-racist policy for first responders in order to mitigate barriers to calling 9-1-1 for an overdose and ultimately reduce overdose deaths.

## Supplementary Information

**Additional file 1.** Good Samaritan Drug Overdose Act Survey. Survey questions asked to respondents either in-person or online.

**Additional file 2.** Supplemental Figure and Table. A concept map of the variables used to inform multivariable model construction and a table comparing the final regression model block generated by complete case analysis vs. multiple imputation.

## Data Availability

The datasets analyzed during the current study are available from the corresponding author on reasonable request.

## Abbreviations

AIC: Akaike information criterion
AOR: Adjusted odds ratio
BC: British Columbia
BCEHS: British Columbia Emergency Health Services
CCA: Complete case analysis
EMS: Emergency medical services
GSDOA: Good Samaritan Drug Overdose Act
MICE: Multiple imputation by chained equation
P2P: Peer2peer Project
PEEP: Professionals for Ethical Engagement of Peers
PWLLE: People with lived and living experience
PWUD: People who use drugs
SES: Socioeconomic status
THN: Take Home Naloxone
US: United States
