# Supplementary material for: Intention to seek emergency medical services during community overdose events in British Columbia, Canada: a cross-sectional survey
Source: Subst Abuse Treat Prev Policy. 2022 Jul 26;17:56. doi: 10.1186/s13011-022-00484-0 (PMC9315848; doi:10.1186/s13011-022-00484-0)
Supplement: Supplementary file 1 — Additional file 1. Good Samaritan Drug Overdose Act Survey. Survey questions asked to respondents either in-person or online. [file 13011_2022_484_MOESM1_ESM.pdf]

Good Samaritan Drug Overdose Act
Knowledge, Attitudes and Experiences Survey (THN Sites)

This survey asks about your thoughts and experiences with overdoses, calling 9-1-1 when an overdose happens, and your knowledge about the Good Samaritan Drug Overdose Act (GSDOA), an Act that aims to encourage seeking medical attention for overdose. We will also be asking you about potential impacts of COVID-19 (corona virus) on responding to overdose. You do not have to answer any questions, including questions about COVID-19 (corona virus), that may be triggering or may cause you discomfort. A "prefer not to answer" option exists for every question. Not answering any question will not disqualify you from participating. No personal identifying information will be collected and your responses will be kept confidential. Support services are available, and a list can be provided by the research coordinator. The survey will take roughly 15 minutes of your time. Please note that you can only complete the survey once.

You are invited to fill out this survey because you are getting a take home naloxone kit and therefore think you are at risk of an opioid overdose and/or likely to see an opioid overdose.

First, we'd like to ask some questions about you:

1. What is your current GENDER identity? (Select all that apply)
❑ Woman ❑ Man ❑ Trans man ❑ Trans woman ❑ Gender non-binary
❑ Other, specify: ______________ ❑ Prefer not to say

2. How old are you? _____________ (years) ❑ Prefer not to say

3. Do you identify yourself as any of the following? (Select one)
❑ First Nations ❑ Métis ❑ Inuit ❑ No, do not identify as Indigenous ❑ Prefer not to say

4. In what region of BC do you live? (Select one)
❑ Northern BC ❑ Interior BC ❑ Vancouver coastal area ❑ Vancouver Island ❑ Fraser area ❑ Prefer not to say

5. Do you live: (Select one)
❑ In a private residence, alone ❑ In a private residence, with someone else
❑ Other residence (hotels, motels, rooming houses, single room occupancy (SRO), shelters, social/supportive housing etc.)
❑ I have no regular place to stay (homeless, couch surf, No Fixed Address)
❑ Other, specify _____________ ❑ Prefer not to say

6. How long has this been your living situation? (Select one)
❑ More than 1 year ❑ 7-12 months ❑ 1-6 months ❑ Less than 1 month ❑ Prefer not to say

7. Are you currently in school? (Select all that apply)
❑ Yes, high school ❑ Yes, post-secondary education ❑ No ❑ Prefer not to say

8. Are you currently employed? (Select all that apply)
❑ Yes, paid volunteer ❑ Yes, part -time ❑ Yes, full-time ❑ No ❑ Prefer not to say

9. Do you have a cellphone? (Select one)
❑ Yes ❑ No ❑ Prefer not to say

10. In the <u>last 6 months</u>, have you felt at risk of experiencing an overdose? (Circle or select one)

Never — Rarely — Sometimes — Often — All the time ❑ Prefer not to say

11. In the <u>last 6 months</u>, have you felt as though it was likely that you would witness an overdose? (Circle one)

Never — Rarely — Sometimes — Often — All the time ❑ Prefer not to say

12. Are you picking up your first Take Home Naloxone Kit? (Select one)
❑ Yes *(skip to #13)* ❑ No ❑ Not picking up a kit *(skip to #13)* ❑ Prefer not to say *(skip to #13)*

12a. <u>If you answered no</u> – why are you picking up a new Take Home Naloxone kit? (Select one)
❒ Used last kit to respond to an overdose ❑ Last kit expired
❒ Lost last kit ❑ Other:_________________
❒ Gave last kit to someone else ❑ Prefer not to say
❒ Last kit was confiscated

Content Warning: In the next part of this survey **we'll be asking about your experiences of overdosing, witnessing overdoses and responding to overdoses,** to get a better understanding of how people respond and what may influence them seeking help. **You are not required to answer** any questions that may cause you to experience discomfort.

13. In the <u>last 6 months</u>, have YOU overdosed (overamped) by accident from using a stimulant (eg. crystal meth or crack)? (Select one)

❑ Yes ❑ No *(skip to #14)* ❑ Don't know *(skip to #14)* ❑ Prefer not to say *(skip to #14)*

13a. <u>If you answered yes</u> – how many times in the last 6 months?___________

14. Have you used opioids in the <u>last 6 months</u>?

❑ Yes ❑ No *(skip to #16)* ❑ Prefer not to say *(skip to #16)*

14a. <u>If you answered yes</u> - how do you take opioids (Select all that apply)**?** ❑ Inject ❑ Smoke ❑ Snort ❑ Swallow

14b. <u>If you answered "*smoke*"</u> – how do you do this?

❑ In foil
❑ In a pipe
❑ Rolled into a cigarette/joint
❑ Other:_____________

15. In the <u>last 6 months</u>, have YOU overdosed (overamped) by accident from using any opioids (eg. fentanyl, heroin)? (Select one)

❑ Yes ❑ No *(skip to #16)* ❑ Don't know *(skip to #16)* ❑ Prefer not to say *(skip to #16)*

15a. <u>If you answered yes</u> – how many times in the last 6 months?___________

16. In the <u>last 6 months</u>, have you SEEN an accidental overdose in someone using any stimulants? (Select one)

❑ Yes ❑ No *(skip to #17)* ❑ Don't know *(skip to #17)* ❑ Prefer not to say *(skip to #17)*

16a. <u>If you answered yes</u> – how many times in the last 6 months?___________

17. In the last 6 months, have you SEEN an accidental overdose in someone using any opioids? (Select one)

❑ Yes ❑ No *(skip to #19)* ❑ Don't know *(skip to #19)* ❑ Prefer not to say *(skip to #19)*

17a. <u>If you answered yes</u> – how many times in the last 6 months?___________

17b. In the last 6 months, who did you see accidentally overdose using any opioids? (Check all that apply)

❒ Someone I didn't know ❑ Partner
❒ Casual friend/acquaintance ❑ Family member
❒ Close friend ❑ Prefer not to say

18. At the last opioid overdose you witnessed, what happened? (Select all that apply)

❑ I left the scene
❑ I gave breaths (no chest compressions)
❑ I gave chest compression (no breaths)
❑ I gave breaths and chest compressions
❑ I gave naloxone/narcan
❑ Someone else gave breaths (no chest compressions)
If so, please circle who: Bystander (friend, stranger...) Staff (overdose prevention site, drop-in…) Paramedic Police
❑ Someone else gave chest compressions (no breaths)
If so, please circle who: Bystander (friend, stranger...) Staff (overdose prevention site, drop-in…) Paramedic Police
❑ Someone else gave breaths and chest compressions
If so, please circle who: Bystander (friend, stranger...) Staff (overdose prevention site, drop-in…) Paramedic Police
❑ Someone else gave naloxone/narcan
If so, please circle who: Bystander (friend, stranger...) Staff (overdose prevention site, drop-in…) Paramedic Police
❑They recovered without any of the above
❑ Prefer not to answer

18a. At the last opioid overdose you witnessed, was 9-1-1 called? (Select one)

❑ Yes *(skip to #18c)* ❑ No ❑ Don't know *(skip to #18c)* ❑ Prefer not to say *(skip to #18c)*

18b. Why was 9-1-1 not called? (Select all that apply)

❑ Didn't have a phone/phone not available
❑ Worried about calling 9-1-1 because I or others have rarely called 9-1-1 before
❑ Situation seemed under control
❑ Worried about family services being notified
❑ Worried about neighbors/landlord knowing about drug use
❑ Worried about mine or someone else's ethnicity and interactions with police/other 9-1-1 responders
❑ Worried about police coming, with no relation to ethnicity. If so, specify why:____

❑ Worried about paramedics or fire services coming, with no relation to ethnicity. If so, specify why:____

❑ Other, please specify:____________________________________

❑ Prefer not to say

18c. At the last opioid overdose you witnessed, did the ambulance come? (Select one)

❑ Yes ❑ No ❑ Don't know ❑ Prefer not to say

18d. At the last opioid overdose you witnessed, did the police come? (Select one)

❑ Yes ❑ No *(skip to #19)* ❑ Don't know *(skip to #19)* ❑ Prefer not to say *(skip to #19)*

18e. What did the police do at the overdose scene? (Select all that apply)

❑ Provided medical assistance (naloxone/Narcan, CPR, etc.)

❑ Watched/bystander role

❑ Zoned off the area

❑ Searched myself and/or others at the scene

❑ Checked for warrants/IDs/red zone restrictions

❑ Made arrests/pressed charges

❑ Other, please specify:____________________________________

❑ Prefer not to say

19. Are you concerned about COVID-19 (corona virus) when you respond to an overdose?

❑ Yes ❑ No *(skip to #20)* ❑ Prefer not to say *(skip to #20)*

19a. If you answered yes – what are your concerns? (Select all that apply)

❑ Proximity to others (e.g. person overdosing, bystanders…)

❑ Giving breaths

❑ Contact with healthcare workers/hospitals

❑ Any other concerns, please specify:____________________________________

❑ Prefer not to say

20. Does COVID-19 (corona virus) make you change the way you respond to an overdose?

❑ Yes ❑ No *(skip to #21)* ❑ Prefer not to say *(skip to #21)*

20a. If you answered yes – how does COVID-19 (corona virus) make you change the way you respond? (Select all that apply)

❑ Won't give breaths

❑ Won't give chest compressions

❑ Will give naloxone sooner

❑ More likely to call 9-1-1

❑ Less likely to call 9-1-1

❑ Any other changes, please specify: _________________

❑ Prefer not to say

Now, we would like to ask some questions about the Good Samaritan Drug Overdose Act:

21. Have you heard about the Good Samaritan Drug Overdose Act? (Select one)

❑ Yes ❑ No *(skip to definition)* ❑ Prefer not to say *(skip to definition)*

22. Have you seen any posters, pamphlets or online material about the GSDOA? (Select one)

❑ Yes ❑ No ❑ Prefer not to say

23. Please indicate your agreement with this statement: The Good Samaritan Drug Overdose Act has encouraged some people to call 9-1-1 in the event of an overdose. (Select one)

❑ Strongly agree ❑ Agree ❑ Neutral ❑ Disagree ❑ Strongly disagree ❑ Don't know

24. Do you believe the GSDOA protects the following people from being arrested for simple possession of substances (small amount of drugs for own use) at the scene of an overdose? (Select all that apply)

a. The person who calls 9-1-1

❑ Yes ❑ No ❑ Don't know ❑ Prefer not to say

b. The person who overdoses

❑ Yes ❑ No ❑ Don't know ❑ Prefer not to say

c. Anyone at the scene of an overdose

❑ Yes ❑ No ❑ Don't know ❑ Prefer not to say

25. Imagine there is an overdose in a public place; 9-1-1 is called and the police come to the scene. Do you think the police can legally arrest a person if they: (Select all that apply)

a. Have a larger amount of drugs on them or items (eg. scale) that may look like they are involved in drug dealing

❑ Yes ❑ No ❑ Don't know ❑ Prefer not to say

b. Are in a red/no-go zone they received for a previous charge that was not simple drug possession (eg. theft)
❑ Yes ❑ No ❑ Don't know ❑ Prefer not to say

c. Have an outstanding warrant for something other than simple drug possession (eg. theft)
❑ Yes ❑ No ❑ Don't know ❑ Prefer not to say

*__Definition__: The **Good Samaritan Drug Overdose Act** was made law in May 2017. It protects the person who overdoses, the person who calls 9-1-1, and anyone else at the scene of an overdose from being arrested for 'simple' possession that means having illegal drugs for their own personal use. It does not protect anyone at an overdose from being arrested for outstanding warrants, controlled substance trafficking or production, or any other serious offense*

26. Based on this description, if you were to witness someone overdose in the future, would you call 9-1-1? (Select one)
❑ Yes *(skip to #27)* ❑ No ❑ Prefer not to say

26a. __If you answered no__ – why not:
- ❑ Don't have a phone
- ❑ Worried about family services being notified
- ❑ Worried about others knowing about drug use
- ❑ Worried about mine or someone else's ethnicity and interactions with police/other 9-1-1 responders
- ❑ Worried about police coming, with no relation to ethnicity. If so, specify why:________________
- ❑ Worried about paramedics or fire services coming, with no relation to ethnicity. If so, specify why:____
- ❑ Other, please specify:_____________________________________
- ❑ Prefer not to say

27. Based on the description, please indicate your level of agreement with the following statement: This act will encourage people to call 9-1-1 in the event of an overdose. (Select one)
❑ Strongly agree ❑ Agree ❑ Neutral ❑ Disagree ❑ Strongly disagree

28. Do you have any other thoughts you'd like to share about the Good Samaritan Drug Overdose Act?

____

____

Thank you for taking the survey!
