# Supplementary material for: Intention to seek emergency medical services during community overdose events in British Columbia, Canada: a cross-sectional survey
Source: Subst Abuse Treat Prev Policy. 2022 Jul 26;17:56. doi: 10.1186/s13011-022-00484-0 (PMC9315848; doi:10.1186/s13011-022-00484-0)
Supplement: Supplementary file 2 — Additional file 2. Supplemental Figure and Table. A concept map of the variables used to inform multivariable model construction and a table comparing the final regression model block generated by complete case analysis vs. multiple imputation. [file 13011_2022_484_MOESM2_ESM.pdf]

Additional File 2

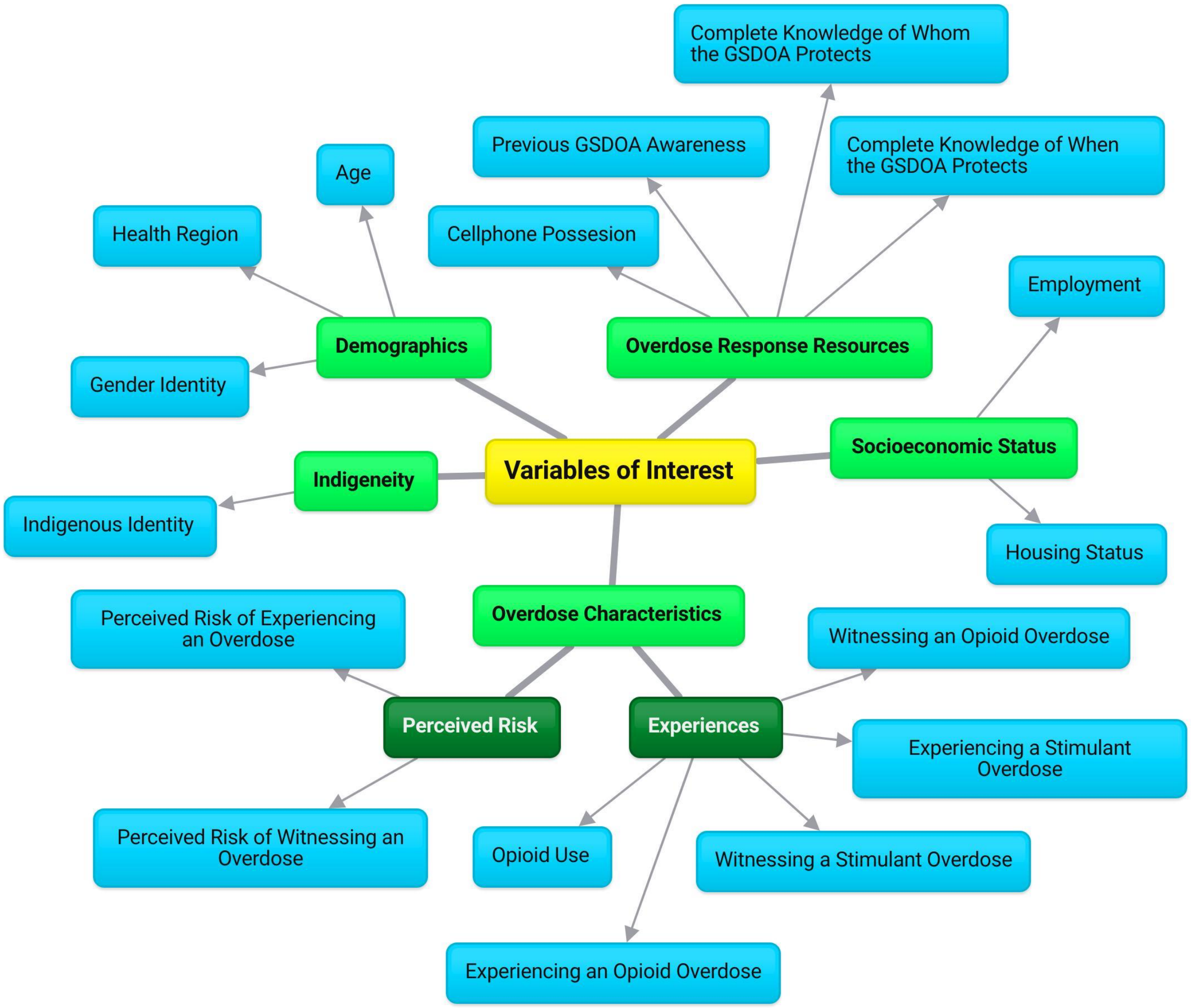

Supplementary Figure 1. Concept map of study variables of interest used to inform the development of a hierarchical regression model. Variables (blue) were assembled into categories (green) and subcategories (dark green) with categories ultimately becoming the "blocks" upon which the regression was built.

Supplementary Table 1. Adjusted odds ratios (AOR) for predictors of intention to call 9-1-1 with multiple imputations by chained equation (MICE).

	**Calling 911 at an OD event**	
	Block 5 (OD Characteristics)[a] AOR (95% CI)	Imputed Block 5 (OD Characteristics)[b] AOR (95% CI)
**Demographic Characteristics**		
Age (years)		
16 – 24	—	—
25 – 34	0.20 (0.04, 0.98) *	0.24 (0.08, 0.76) *
35 – 44	0.24 (0.05, 1.18)	0.33 (0.11, 1.06)
45 – 54	0.55 (0.10, 3.07)	0.78 (0.20, 3.03)
55 +	0.17 (0.03, 0.90) *	0.40 (0.12, 1.36)
Gender		
Cis man	—	—
Cis woman	3.37 (1.19, 9.50) *	1.64 (0.76, 3.54)
Indigenous Identity[c]		
Non-indigenous	—	—
Indigenous	0.64 (0.25, 1.64)	0.84 (0.40, 1.80)
**SES Factors**		
Housing Status		
Private - Alone	—	—
Private - With others	4.96 (1.21, 20.29) *	4.76 (1.69, 13.44) **
Supportive/Unstable Housing	3.96 (1.31, 11.98) *	5.20 (2.29, 11.78) ***
Homeless	2.39 (0.58, 9.82)	3.42 (1.15, 10.16) *
Employment Status		
Not employed	—	—
Employed	0.46 (0.18, 1.18)	0.44 (0.21, 0.91) *
**Overdose Response Resources**		
Previous awareness of GSDOA		
Unaware	—	—
Aware	4.16 (1.62, 10.67) **	2.28 (1.08, 4.79) *
**Overdose Characteristics**		
Stimulant OD		
No	—	—
Yes	0.24 (0.09, 0.65) **	0.46 (0.19, 1.13)

Note: *p<0.05, **p<0.01, ***p<0.001.

[a] Final unimputed model is based on N = 327 observations after exclusion of "unknown" responses for each variable.

[b] Final imputed model is based on N = 493 observations after "unknown" responses were imputed via MICE (54).

[d] The authors recognize that Indigenous identity is often a proxy for factors like intergenerational trauma, systemic racism, and socioeconomic status.

[d] "Never" = "Never"; "Ever" = "Rarely/sometimes/often/all the time"
